# Low-dose Dasatinib Ameliorates Hypertrophic Cardiomyopathy in Noonan Syndrome with Multiple Lentigines

**DOI:** 10.1007/s10557-021-07169-z

**Published:** 2021-03-10

**Authors:** Jae-Sung Yi, Sravan Perla, Yan Huang, Kana Mizuno, Frank J. Giordano, Alexander A. Vinks, Anton M. Bennett

**Affiliations:** 1 Department of Pharmacology, Yale University School of Medicine, New Haven, CT 06520 USA; 2 Department of Internal Medicine, Yale University School of Medicine, New Haven, CT 06520 USA; 3 Division of Clinical Pharmacology, Cincinnati Children’s Hospital Medical Center, Cincinnati, OH USA; 4 Department of Pediatrics, University of Cincinnati, College of Medicine, Cincinnati, OH USA; 5 Program in Integrative Cell Signaling and Neurobiology of Metabolism, Yale University School of Medicine, New Haven, CT 06520 USA

**Keywords:** Tyrosine kinase inhibitor, Hypertrophic cardiomyopathy, RASopathy, Noonan syndrome with multiple lentigines, Protein tyrosine phosphatases

## Abstract

**Purpose:**

Noonan syndrome with multiple lentigines (NSML) is an autosomal dominant disorder presenting with hypertrophic cardiomyopathy (HCM). Up to 85% of NSML cases are caused by mutations in the *PTPN11* gene that encodes for the Src homology 2 (SH2) domain-containing protein tyrosine phosphatase 2 (SHP2). We previously showed that low-dose dasatinib protects from the development of cardiac fibrosis in a mouse model of NSML harboring a *Ptpn11*^*Y279C*^ mutation. This study is performed to determine the pharmacokinetic (PK) and pharmacodynamic (PD) properties of a low-dose of dasatinib in NSML mice and to determine its effectiveness in ameliorating the development of HCM.

**Methods:**

Dasatinib was administered intraperitoneally into NSML mice with doses ranging from 0.05 to 0.5 mg/kg. PK parameters of dasatinib in NSML mice were determined. PD parameters were obtained for biochemical analyses from heart tissue. Dasatinib-treated NSML mice (0.1 mg/kg) were subjected to echocardiography and assessment of markers of HCM by qRT-PCR. Transcriptome analysis was performed from the heart tissue of low-dose dasatinib-treated mice.

**Results:**

Low-dose dasatinib exhibited PK properties that were linear across doses in NSML mice. Dasatinib treatment of between 0.05 and 0.5 mg/kg in NSML mice yielded an exposure-dependent inhibition of c-Src and PZR tyrosyl phosphorylation and inhibited AKT phosphorylation. We found that doses as low as 0.1 mg/kg of dasatinib prevented HCM in NSML mice. Transcriptome analysis identified differentially expressed HCM-associated genes in the heart of NSML mice that were reverted to wild type levels by low-dose dasatinib administration.

**Conclusion:**

These data demonstrate that low-dose dasatinib exhibits desirable therapeutic PK properties that is sufficient for effective target engagement to ameliorate HCM progression in NSML mice. These data demonstrate that low-dose dasatinib treatment may be an effective therapy against HCM in NSML patients.

**Supplementary Information:**

The online version contains supplementary material available at 10.1007/s10557-021-07169-z.

## Introduction

Protein tyrosyl phosphorylation is a reversible post-translational modification that controls developmental and post-developmental functions [1]. The balance of protein tyrosyl phosphorylation is determined by the opposing activities of protein tyrosine kinases (PTKs) and protein tyrosine phosphatases (PTPs). It is established that imbalance between the activities of PTKs and PTPs leads to aberrant regulation of protein tyrosyl phosphorylation that causes a variety of human diseases [2]. The Src homology 2 (SH2) domain-containing protein tyrosine phosphatase 2 (SHP2), which is encoded by the *PTPN11* gene, acts as a positive regulator in signal transduction pathways, such as the Ras-mitogen-activated protein kinase (MAPK) and the phosphatidylinositol 3’-kinase (PI3K)-AKT pathways [3]. Genetic studies have revealed that mutations in SHP2 are associated with the pathogenesis of human diseases that includes solid tumors, leukemia and congenital heart diseases (CHD) [3–5].

CHD represents the leading form of birth defect and cause of death among newborns, presenting with various valvuloseptal and myocardial abnormalities [6, 7]. Although the causes of CHD among newborns are unknown, several genetic factors, such as chromosomal abnormalities and syndromic gene mutations, are associated with CHD. Around 20% of genetic syndromes present with cardiac disease in infants [8]. A striking example of genetic syndromes are the RASopathies which, represent mutations in the Ras-MAPK pathway, leading to an array of birth defects including CHD [9]. Mutations in SHP2, encoded by the *PTPN11* gene, cause Noonan syndrome (NS) and Noonan syndrome with multiple lentigines (NSML) [5, 10, 11]. NS and NSML patients exhibit similar, but non-overlapping clinical features, despite the fact that NS- and NSML-associated *PTPN11* mutations give rise to opposing effects on SHP2 catalysis [10–12]. The overlapping clinical features between NS and NSML, which include the presentation of CHD, have been suggested to be a result of disruption of the closed conformation of SHP2 [13–15]. The open conformation of SHP2 exhibits increased propensity to engage in protein-protein interactions [16]. Since both NS- and NSML-associated *PTPN11* mutations assume this open conformation, it does suggest that at least some component of this altered structure underlies common features between the two diseases. NS-associated *PTPN11* mutations typically present with pulmonic valve stenosis and to a much lesser extent hypertrophic cardiomyopathy (HCM) [17]. In contrast, 85–90% of NSML cases present with HCM [18]. Given the possibility that both NS- and NSML-associated *PTPN11* mutations might function more like adapter proteins, we sought to identify upstream binding proteins that these SHP2 mutations might interact promiscuously with. We identified the protein zero-related (PZR), a cell surface glycoprotein, as a major NS-associated SHP2 mutant binding protein in NS mutant expressing cells, embryos and in heart lysates derived from NS mice (*Ptpn11*^*D61G/+*^) [19, 20]. Consistent with the notion that the open conformation of SHP2-associated mutations might underlie some of the common mechanisms between NS and NSML, in a mouse model of NS (*Ptpn11*^*D61G/+*^) and NSML (*Ptpn11*^*Y279C/+*^), PZR is hypertyrosyl phosphorylated in the heart [20]. When PZR tyrosyl phosphorylation is disrupted in NS mice using a low dose of dasatinib, a Bcr-Abl and Src family kinase inhibitor, the interaction between PZR and SHP2 is prevented and this correlated with improved cardiac function in NS mice. In addition, low-dose dasatinib reduced the expression of molecular markers of HCM in NS and NSML mice [21]. These results suggested that promiscuous PZR/SHP2 interactions in NS, and possibly NSML, promote CHD. Indeed, this assertion is supported by our recent observation that when a mutant of PZR that fails to bind SHP2 is introduced in to NSML mice, the development of HCM is completely prevented [22]. Notably, activation of AKT in NSML mice which is involved in promoting cardiomyocyte growth was inhibited in the hearts of NSML mice-bearing the PZR mutant that fails to bind SHP2 [22]. Thus, PZR/SHP2 interactions, which are sensitive to a low dose of dasatinib, appear to be involved in the progression of HCM. However, whether low-dose dasatinib prevents the development of HCM in NSML mice remains to be demonstrated formally.

Given the important role of AKT in heart growth and in pathophysiological signaling in HCM [23–25], strategies to inhibit AKT for the treatment of HCM in NSML has been investigated. Treatment of NSML mice with rapamycin has been shown to inhibit AKT and prevent the development of HCM [26–28]. Additionally, in one case, the rapamycin analog, Everolimus, which inhibits the mTOR/AKT pathway was found to slow the progression of HCM in a NSML patient [29]. These results suggest that targeting AKT might be a viable treatment of HCM in NSML patients. The finding that genetic inhibition of PZR/SHP2 interactions inhibits AKT activation and prevents the development of HCM in NSML mice prompted the question as to whether pharmacological inhibition of this interaction could also ameliorate AKT activity and HCM progression in NSML mice [22]. Here, we report that treatment of NSML mice with a low dose of dasatinib, an FDA-approved chemotherapeutic drug, inhibited AKT activity and prevented the development of HCM in NSML mice. Low-dose dasatinib administration into NSML mice displayed linear pharmacokinetic and pharmacodynamic properties and demonstrated target engagement by inhibiting c-Src and PZR tyrosyl phosphorylation in an exposure-dependent manner in the heart of NSML mice. Taken together, these results demonstrate that low-dose dasatinib represents a viable strategy for the treatment of HCM in NSML.

## Methods

### Animal Handling

NSML (*Ptpn11*^*Y279C/+*^) mice were obtained from The Jackson Laboratory (Stock number: 026759). NSML male mice were crossed with C57BL/6J female, and offspring were genotyped by PCR for the *Ptpn11* Y279C allele. Dasatinib (Biovision) was suspended in vehicle (1% DMSO in citrate buffer). Dasatinib was injected daily (i.p.) into mice at the age of 8 weeks for 4 weeks for the PK study and echocardiography, and at 14 weeks of age for 4 weeks for RNA-seq analysis. Animals were housed and cared for in facilities run by the Division of Animal Care and were routinely monitored by the veterinary staffs. Animal handling was approved by Yale University Institutional Animal Care and Use Committee.

### Antibodies, Chemicals, Cell lines, and Plasmids

The following antibodies were used for immunoblotting. Rabbit monoclonal phospho-PZR (Y241; D6F9, #8131), rabbit monoclonal phospho-PZR (Y263; D6A5, #8088), rabbit polyclonal phospho-Src (Y416; #2101), rabbit polyclonal Src (#2108); rabbit polyclonal phospho-AKT (S473; #9271), mouse monoclonal AKT (C73H10, #2938), rabbit polyclonal phospho-ERK1/2 (T202/Y204; #9101), mouse monoclonal ERK (3A7, #9107) antibodies were purchased from Cell Signaling Technology. Rabbit polyclonal PZR antibody (105-6) was generously provided by Z. J. Zhao. Dasatinib (#1586-100) was purchased from Biovision.

### Pharmacokinetic Analysis

Mice were administered vehicle, or dasatinib at a dose of 0.05, 0.1, 0.25, or 0.5 mg/kg intraperitoneally (i.p.). Each dosed cohort consisted of six animals. One blood sample was collected from each animal at the following times: 1, 2, 3, 4, 6, and 8-h post-dose. Dasatinib PK parameters for each dose cohort were estimated using Bayesian estimation with a two-compartmental dasatinib PK model. (MW\Pharm version 3.82; Mediware, Prague, Czech Republic). The PK model parameters and their distributions were generated using rich post-dose PK data in a NSML mouse model and were evaluated against mouse PK data reported by Luo et al. [30]. AUC estimates for each dose group were generated by dividing dose by the clearance estimate.

### Immunoblotting

Heart tissue was lysed on ice in lysis buffer (25 mM Tris-HCl, pH 7.4, 136 mM NaCl, 1 mM CaCl_2_, 1 mM MgCl_2_, 1% Nonidet P-40, 1 mM Na_3_VO_4_, 10 mM NaF, 1 mM benzamidine, 1 mM PMSF, 1 μg/ml pepstatin A, 5 μg/ml aprotinin, and 5 μg/ml leupeptin). Tissue lysates were incubated at 4 °C for 30 min and clarified by centrifugation at 14,000 rpm at 4 °C for 10 min. Protein concentration was determined using the BCA reagent and lysates were subjected to SDS-PAGE and immunoblotting. The sites of antibody binding were visualized and were quantified using the Odyssey CLx Imaging System (LI-COR Bioscience).

### Exposure-Response Analysis

Src tyrosyl phosphorylation (Y416) and PZR tyrosyl phosphorylation (Y242 and Y264) data were used as pharmacodynamic markers and key elements causing HCM in the exposure-response analysis. AUC estimates were used as the dasatinib exposure data. Data were fitted to an inhibitory sigmoidal *E*_max_ model with the following assumption: (1) pSrc/Src converges to zero and (2) p-PZR/PZR converges to the wild-type level. The exposure-response relationship was evaluated by fitting with an Inhibitory *E*_max_ model as follows: *E* = *E*_0_ – (*I*_max_ + AUC^γ^)/(IAUC_50_^γ^ + AUC^γ^), where *E*_0_ is the baseline effect, *I*_max_ is maximum inhibitory effect, and IAUC_50_ is the AUC at which 50% of *I*_max_ is produced. The corresponding dose to achieve IAUC_50_ was calculated by using the following equation: IAUC_50_ x mean estimated CL.

### Echocardiography

Mice were anesthetized in a sealed plastic chamber with 1% isofurane in oxygen until immobile, and then were transferred onto a heated procedure board (37 °C). Animals were kept anesthetized with 1% isoflurane supplied by a nose cone connected to the vaporizer during the entire procedure. The scan head was placed on the chest of the mouse and stable image signals (both B mode and M mode) were acquired and data analyzed with Vevo 770 (VisualSonics).

### RNA Extraction and Quantitative Real-time PCR Analysis

RNA was isolated from mice heart using an RNeasy kit (#74104; Qiagen). A total of 1 μg RNA was reverse transcribed to generate cDNA using a reverse transcriptase PCR kit (#4368814; Applied Biosystems). Real-time quantitative PCR was carried out in triplicate using the Applied Biosytems 7500 Fast real-time PCR system and PowerUp SYBR green master mix (#A25742; Applied Biosystems) with primer pairs listed in Supplementary Table 1. All relative gene expression levels were analyzed using the ΔΔC_T_ method and normalized to 18S rRNA expression.

### RNA-seq and Data Analysis

RNA-seq analysis was performed as described previously [22]. Total RNA isolated from the heart of mice were purified and amplified to sequencing libraries. Samples were sequenced on an Illumina NovaSeq according to Illumina protocols. Data generated during sequencing runs were simultaneously transferred to the YCGA high-performance computing cluster. Signal intensities were converted to individual base calls during a run using the system’s Real Time Analysis (RTA) software. Base calls were transferred from the machine’s dedicated personal computer to the Yale High Performance Computing cluster via a 1 Gigabit network mount for downstream analysis. The RNAseq and statistical analysis was performed using Partek Flow Genomic Analysis software build version 8.0.19.1125 (Partek Inc.). Paired-end reads were trimmed and aligned to the Genome Reference Consortium Mouse Build 38 (mm10) with the STAR alignment tool (ver. 2.6.1d). Total counts per gene were quantified and normalized to identify differentially expressed genes (DEGs). List of DEGs were generated by DESeq2. Qlucore Omics Explorer 3.5 (Qlucore AB) was used for PCA (*p* = 0.0001) of log_2_ transformed of global expression values, heatmap generation, and hierarchical clustering (*p* ≤ 0.01). Ingenuity Pathway Analysis (QIAGEN), IPA software (ver. 10–14) was used to do gene ontology (GO) enrichment analysis and to identify top upstream regulators, top diseases, and biological functions.

### Statistical Analysis

Sample size for animal studies was not estimated and randomization was not applied. The investigators were blinded during echocardiography experiments and outcome assessment. Statistical analysis and graphing were performed using GraphPad Prism 8 software. All data represent the means ± standard errors of the means (SEM). For *p* value determinations, we used ANOVA with multiple comparison, two-stage linear step-up procedure of Benjamini, Krieger, and Yekutieli.

## Results

### Pharmacokinetics and Pharmacodynamics of Dasatinib in NSML Mice

*Ptpn11*-associated NSML mice (*Ptpn11*^*Y279C/+*^) progressively develop hypertrophic cardiomyopathy (HCM) that is apparent by 12 weeks of age [27]. Our previous report showed that postnatal administration of a low dose of dasatinib normalized the expression of the molecular markers of HCM and reduced myocardial fibrosis in NSML mice [21]. Allometric calculations of the dose of dasatinib that effectively reversed NSML-associated increases of HCM markers and myocardial fibrosis were found to be up to 200-fold lower than that used for the treatment of chronic myeloid leukemia (CML) in human [21]. Here, we set out to characterize the pharmacokinetic (PK) and pharmacodynamic (PD) parameters to better understand dasatinib target engagement and to further evaluate the effects of low-dose dasatinib in the progression of HCM in NSML mice.

**Fig. 1:**
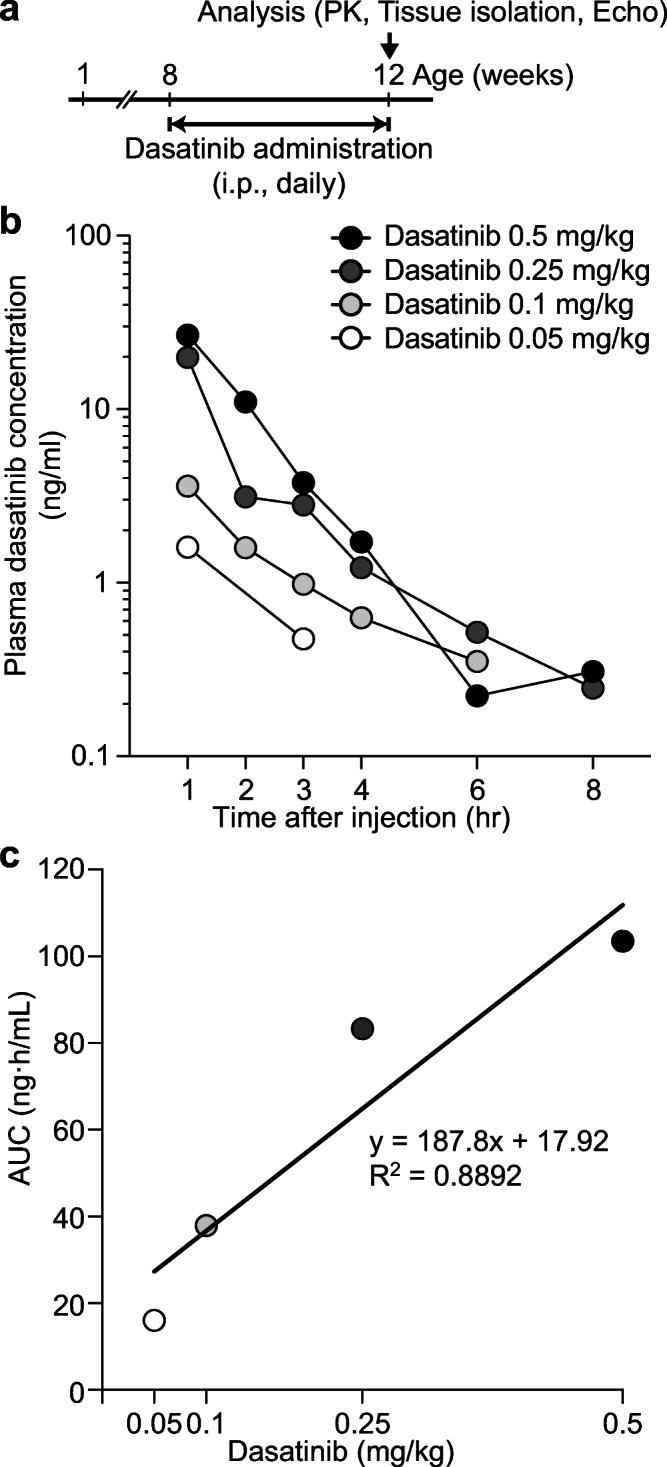
Plasma pharmacokinetics of low-dose dasatinib in NSML mice. **a** Schematic of low-dose dasatinib administration. Vehicle or dasatinib (0.05, 0.1, 0.25, or 0.5 mg/kg) was intraperitoneally (i.p., daily) injected into 8-week-old wild-type (WT) or NSML mice for 4 weeks. At the age of 12 weeks, animals were subjected to PK study and heart tissues were isolated. Plasma pharmacokinetics of low-dose dasatinib in NSML mice. After dasatinib i.p. injection, mice were bled by cardiac puncture at the indicated time points. **b** Concentrations of dasatinib were analyzed by liquid chromatography/mass spectrometry. **c** Correlation between dasatinib dose and the area under the plasma dasatinib concentration-time curve estimates (AUC) was plotted

We found that higher doses (0.25 mg/kg and 0.5 mg/kg) of dasatinib injection to neonatal mice increases lethality and reduces body weight. However, 8-week-old mice were viable and did not show signs of reduced body weight during dasatinib administration (0.05–0.5 mg/kg). We administered 0.05 to 0.5 mg/kg of dasatinib daily i.p. to 8-week-old NSML mice for 4 weeks (Fig. 1a). At the age of 12 weeks, serum was collected from those mice at 1, 2, 3, 4, 6, and 8 h after dasatinib administration. Plasma concentration of dasatinib was then determined by high-performance liquid chromatography/mass spectrometry (Fig. 1b). PK parameters of each group were estimated using a two-compartment model (Table 1). The area under the plasma concentration-time curve (AUC) which represents dasatinib exposure levels were 16.0, 37.9, 83.3, and 103.5 ng·h/mL in mice receiving 0.05, 0.1, 0.25, and 0.5 mg/kg of dasatinib, respectively (Table 1). These results showed that the AUC was linearly increased with dasatinib dose (*R*^2^ = 0.8892) (Fig. 1c).

**Table 1:** Pharmacokinetic parameter estimates in dasatinib-treated NSML mice. Dasatinib PK parameters were estimated using PK model-informed Bayesian estimation. CL, dasatinib clearance from the central compartment; Vc, volume of distribution of the central compartment; Q, inter-compartmental clearance; Vp, volume of distribution of the peripheral compartment; AUC, area under concentration-time curve calculated by dividing dose by CL

	Dasatinib (mg/kg)			
	0.05	0.1	0.25	0.5
CL (ml/h)	74.1	71.2	70.0	114.2
Vc (ml)	21.4	21.0	32.9	89.5
Q (ml/h)	16.2	15.2	11.5	11.1
Vp (ml)	29.2	33.0	22.4	25.0
AUC (ng·h/mL)	16.0	37.9	83.3	103.5

### Low-dose Dasatinib Inhibits PZR Tyrosyl Phosphorylation and Signaling in the Hearts of NSML Mice

SHP2 directly binds through its SH2 domains to tyrosyl phosphorylated PZR and in the hearts of NSML mice PZR is hypertyrosyl phosphorylated [19, 31, 32]. Recently, we demonstrated that a mutant of PZR that fails to become tyrosyl phosphorylated and bind SHP2, when introduced in to NSML mice prevents the development of HCM [22]. These results demonstrated that PZR tyrosyl phosphorylation and by inference SHP2 binding is critical for the development of HCM in NSML mice. Although low-dose dasatinib inhibits PZR tyrosyl phosphorylation and rescues HCM and cardiac function in a mouse model of Noonan syndrome (*Ptpn11*^*D61G/+*^) [21], the effectiveness of dasatinib in preventing PZR hypertyrosyl phosphorylation in the hearts of NSML mice and whether this correlates with prevention of HCM is unknown.

**Fig. 2:**
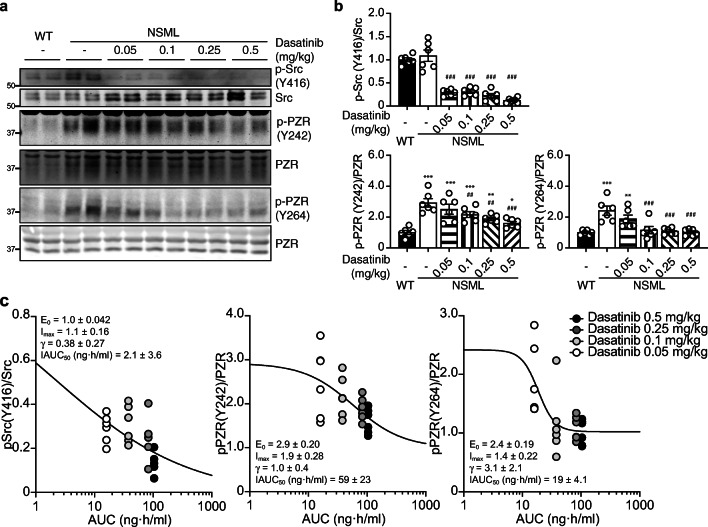
Low-dose dasatinib blocks Src and PZR phosphorylation in the hearts of NSML mice. Heart tissue was isolated from vehicle- or dasatinib- (0.05, 0.1, 0.25, or 0.5 mg/kg) treated wild-type (WT) and NSML mice. **a** Heart lysates were immunoblotted with anti-p-Src (Y416), Src, p-PZR (Y242), p-PZR (Y264), PZR antibodies. **b** The phosphorylation of Src and PZR (Y242 and Y264) were quantified (*n* = 6). Data represent mean ± SEM, *p < 0.05; **p < 0.01; and ***p < 0.001 denotes significance compared with the vehicle-treated WT mice. ##*p* < 0.01 and ###*p* < 0.001 denotes significance compared with the vehicle-treated NSML mice. 1-way ANOVA with 2-stage linear step-up procedure of Benjamini, Krieger, and Yekutieli correction for multiple comparisons. **c** The relationship between the area under the plasma dasatinib concentration-time curve (AUC) from Table 1 and the densitometry of Src phosphorylation at Y416 and PZR tyrosyl phosphorylation at Y242 and Y264 were plotted. Data were fitted to an inhibitory sigmoidal *E*_max_ model (solid line). *E*_0_, the baseline effect; *I*_max_, the maximum inhibitory effect; *γ*, the hill coefficient; IAUC_50_, the AUC at which 50% of *I*_max_ is achieved

**Fig. 3:**
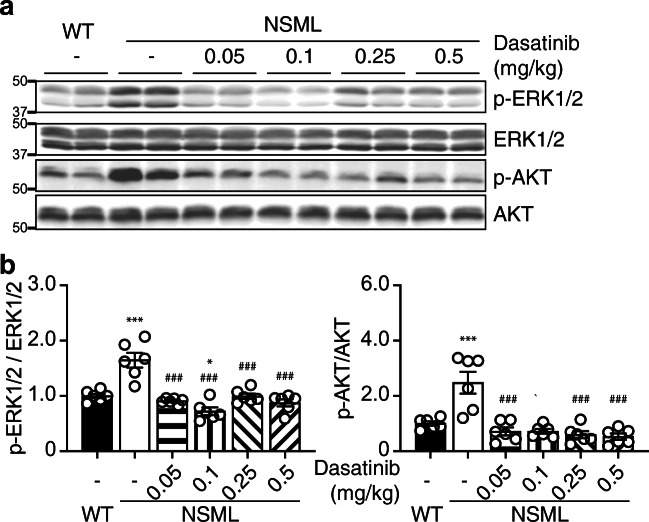
Low-dose dasatinib inhibits HCM signaling in the hearts of NSML mice. Heart tissue was isolated from vehicle- or dasatinib-treated (0.05, 0.1, 0.25, or 0.5 mg/kg) WT and NSML mice. **a** Heart lysates were immunoblotted with anti-p-ERK1/2, ERK1/2, p-AKT (S473), AKT antibodies. **b** The phosphorylation of ERK1/2 and AKT were quantified (*n* = 6). Data represent mean ± SEM, *p < 0.05; and ***p < 0.001 denotes significance compared with the vehicle treated WT mice. ###*p* < 0.001 denotes significance compared with the vehicle-treated NSML mice. 1-way ANOVA with 2-stage linear step-up procedure of Benjamini, Krieger, and Yekutieli correction for multiple comparisons

We first set out to assess the effects of low dose dasatinib on signaling in the hearts of NSML mice. Heart lysates prepared from the ventricles of NSML mice following dasatinib administration (Fig. 1a) showed significant inhibition of c-Src phosphorylation (Fig. 2a, b). We observed that PZR hyper tyrosyl phosphorylation at Y242 and Y264 in heart lysates of NSML mice was inhibited by dasatinib at doses as low as 0.05 mg/kg (Fig. 2a, b). We assessed the response relationship of dasatinib exposure across the dose range of 0, 0.05, 0.1, 0.25, and 0.5 mg/kg on c-Src and PZR tyrosyl phosphorylation. We found the half maximal inhibitory AUC (IAUC_50_) for inhibiting c-Src Y416, PZR Y242, and PZR Y264 phosphorylation were 2.1 ± 3.6, 59 ± 23, and 19 ± 4.1 ng·h/mL, respectively (Fig. 2c). These data are consistent with the linear PK parameters of dasatinib and demonstrate that doses as low as 0.05 mg/kg inhibit c-Src activity and PZR phosphorylation. Next, we assessed the phosphorylation status of downstream targets that have been implicated in cardiac hypertrophy and growth. We found that ERK1/2 and AKT phosphorylation were significantly reduced at dasatinib doses as low as 0.05 mg/kg in heart lysates of dasatinib-treated NSML mice (Fig. 3a, b). Collectively, these data demonstrate that low-dose dasatinib attenuates c-Src and PZR hyper tyrosyl phosphorylation and inhibits ERK and AKT phosphorylation in heart lysates derived from NSML mice.

### Effects of Low-dose Dasatinib in NSML-associated Cardiomyopathy-related Gene Expression

**Fig. 4:**
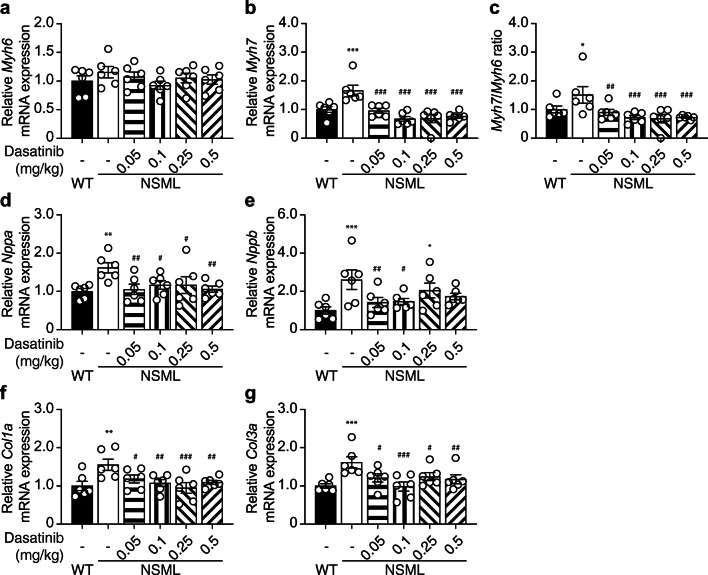
Low-dose dasatinib attenuates the expression of hypertrophic cardiomyopathy genes in NSML mice. Total RNA was isolated from the heart of 12-week-old WT and NSML mice after vehicle or dasatinib (0.05, 0.1, 0.25, or 0.5 mg/kg) administration. The relative mRNA expression levels of **a** α-myosin heavy chain (*Myh6*), **b** β-myosin heavy chain (*Myh7*), **c** ratio of *Myh7* to *Myh6*, natriuretic peptides, **d** *Nppa*, **e** *Nppb*, and fibrosis markers, **f** *Col1a*, and **g** *Col3a* were measured by quantitative reverse transcription PCR (qRT-PCR) (*n* = 6). Data represent mean ± SEM, *p < 0.05; **p < 0.01, and ***p < 0.001 denotes significance compared with the vehicle-treated WT mice. #*p* < 0.05; ##*p* < 0.01, and ###*p* < 0.001 denotes significance compared with the vehicle-treated NSML mice. 1-way ANOVA with 2-stage linear step-up procedure of Benjamini, Krieger, and Yekutieli correction for multiple comparisons

The development of pathophysiological HCM is preceded by re-expression of sarcomeric myosin heavy chain 7 (*Myh7*) genes. Consistent with this, NSML mice which progress to HCM by 16 weeks of age have been shown to re-express *Myh7* [27]. To determine whether in NSML mice dasatinib affects HCM development, we measured the expression of myosin heavy chain 6 (*Myh6*) and *Myh7* in the hearts of vehicle and low-dose dasatinib-treated NSML mice. This analysis revealed increased re-expression of *Myh7* and the increase in ratio of *Myh7/Myh6* was completely prevented in the heart of dasatinib-treated NSML mice as compared with vehicle-treated NSML mice (Fig. 4a–c). We also found that increased expression of cardiomyopathy markers, natriuretic peptide A (*Nppa*) and natriuretic peptide B (*Nppb*) seen in vehicle-treated NSML mice were significantly inhibited in dasatinib-treated NSML mice (Fig. 4d, e). These results demonstrate that low-dose dasatinib treatment of NSML mice prevents the development of HCM at the molecular level. The development of HCM is accompanied later on by myocardial fibrosis. We had shown previously that low-dose dasatinib treatment of NSML mice prevented the development of myocardial fibrosis in hypertrophic hearts [21]. In line with these previous observations, NSML mice treated with low-dose dasatinib similarly showed reduced expression of collagen 1a (*Col1a*) and collagen 3a (*Col3a*) in the heart of NSML mice (Fig. 4f, g). Collectively, these results demonstrate the effectiveness of low-dose dasatinib to inhibit molecular markers of HCM and subsequently the development of secondary fibrotic manifestations in the hearts of NSML mice.

### Low-dose Dasatinib Ameliorates HCM in NSML Mice

Our data indicates that the AKT pathway and the molecular markers that accompany HCM are reduced by low-dose dasatinib treatment of NSML mice strongly suggesting that this contributes to the reversal of HCM development. To determine if this is the case, male NSML mice were treated with either vehicle or a low dose of dasatinib (Fig. 1a). Vehicle-treated NSML mice developed HCM by 12 weeks of age as indicated by a significant increase in heart weight (HW) to body weight (BW) ratios (HW/BW) and heart weight to tibia length (TL) ratios (HW/TL) as compared with vehicle-treated WT mice (Table 2). NSML mice treated with 0.05 mg/kg dasatinib showed a trend towards reduced HW/BW and HW/TL, while 0.1 mg/kg of dasatinib significantly reduced HW/BW and HW/TL as compared with vehicle-treated WT mice (Table 2). Although the higher doses of dasatinib-treated NSML mice (0.25 mg/kg and 0.5 mg/kg) showed reduced HW/BW and HW/TL, the reduction did not achieve statistical significance. This could potentially be attributed to the observation that these higher doses of dasatinib were less well tolerated in NSML mice. Nevertheless, these results are consistent with the inhibitory effects of low-dose dasatinib at the biochemical and molecular levels culminating in the prevention of HCM in NSML mice.

**Table 2:** Anatomic parameters of low-dose dasatinib-treated NSML mice. Body weight (BW), heart weight (HW), the ratio of HW to BW (HW/BW), tibia length (TL), and the ratio of HW to TL (HW/TL) were measured from vehicle or dasatinib-treated (0.05, 0.1, 0.25, or 0.5 mg/kg) males at the age of 12-week-old WT and NSML mice (*n* = 6). Data represent mean ± SEM, *p* value denotes significance compared to the vehicle-treated NSML mice. 1-way ANOVA with 2-stage linear step-up procedure of Benjamini, Krieger, and Yekutieli correction for multiple comparisons

	WT vehicle	NSML vehicle	NSML dasatinib (mg/kg)			
			0.05	0.1	0.25	0.5
BW (g)	26.57 ± 0.39(p = 0.052)	23.82 ± 0.87	23.73 ± 0.88 (p = 0.947)	26.98 ± 1.14(p = 0.027)	23.31 ± 0.96 (p = 0.71)	23.63 ± 1.28 (p = 0.894)
HW (mg)	108.7 ± 1.4(p = 0.831)	110.0 ± 3.5	103.5 ± 2.9 (p = 0.301)	111.7 ± 5.4 (p = 0.789)	105.0 ± 5.6 (p = 0.425)	102.8 ± 5.7 (p = 0.255)
HW/BW (mg/g)	4.09 ± 0.06(p = 0.003)	4.64 ± 0.18	4.37 ± 0.08 (p = 0.117)	4.13 ± 0.07 (p = 0.005)	4.51 ± 0.17 (p = 0.429)	4.35 ± 0.07 (p = 0.094)
TL (mm)	19.39 ± 0.41(p = 0.071)	17.41 ± 0.82	17.27 ± 0.65 (p = 0.895)	19.67 ± 0.81 (p = 0.041)	17.06 ± 0.94 (p = 0.745)	17.15 ± 0.76 (p = 0.812)
HW/TL (mg/mm)	5.62 ± 0.16 (p = 0.019)	6.38 ± 0.35	6.02 ± 0.16 (p = 0.244)	5.68 ± 0.17 (p = 0.03)	6.17 ± 0.21 (p = 0.51)	6.00 ± 0.20 (p = 0.223)

**Fig. 5:**
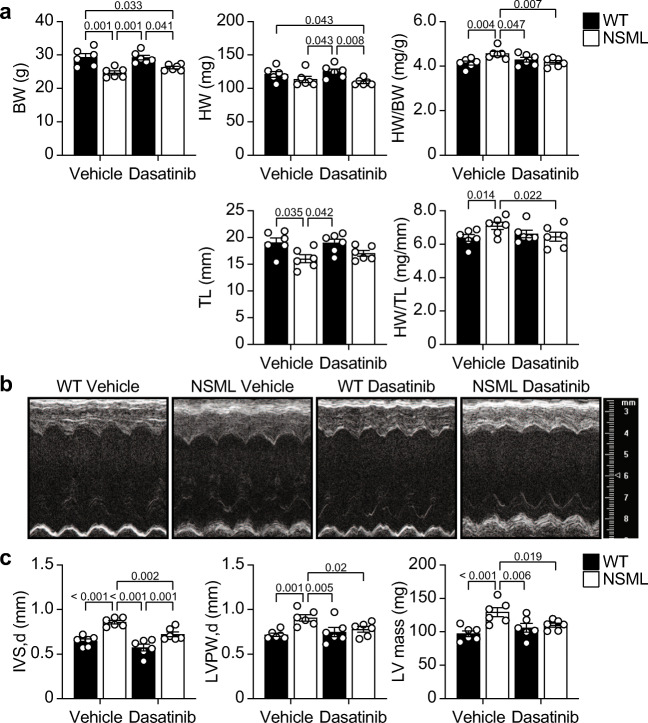
Echocardiographic analysis of NSML mice after low-dose dasatinib administration. WT and NSML mice were treated daily with vehicle or 0.1 mg/kg dasatinib (i.p.) from the age of 8 weeks for 4 weeks. Echocardiography was performed at the age of 12 weeks. **a** Body weight (BW), heart weight (HW), the ratio of HW to BW (HW/BW), tibia length (TL), and the ratio of HW to TL (HW/TL) were measured from vehicle or 0.1 mg/kg of dasatinib-treated WT and NSML mice. **b** Representative echocardiographic images of vehicle- or dasatinib-treated WT and NSML mice. **c** Left ventricular posterior wall thickness in diastolic phase (LVPW, d), interventricular septum wall thickness in diastolic phase (IVS, d) were measured from echocardiograms. The left ventricle mass (LV mass) was calculated from echocardiograms (*n* = 6). Data represent mean ± SEM, 2-way ANOVA with 2-stage linear step-up procedure of Benjamini, Krieger, and Yekutieli correction for multiple comparisons.

To provide further evidence that a low dose of dasatinib has an inhibitory effect on the progression of HCM in NSML mice, we next measured the echocardiographic dimensions of the hearts of low-dose dasatinib-treated NSML mice. We selected a dose of 0.1 mg/kg of dasatinib because it was the lowest effective dose that showed significant inhibition of HCM at the biochemical, genetic, and anatomic levels in the hearts of NSML mice (Figs. 3, 4 and Table 2) and because this dose did not exhibit any observable adverse effects [21]. Male NSML mice at 8 weeks of age were treated with 0.1 mg/kg dasatinib for 4 weeks (Fig. 1a) and echocardiography was performed. We confirmed the significant increase in HW/BW and HW/TL in vehicle-treated NSML mice were normalized in low-dose dasatinib-treated NSML mice (Fig. 5a). We found that increased diastolic interventricular septum wall thickness (IVS, d), diastolic left ventricular posterior wall thickness (LVPW, d), and calculated left ventricular mass (LV mass) in NSML mice were all significantly reduced in dasatinib-treated NSML mice (Fig. 5b, c and Supplementary Table 2). Collectively, these data show that low-dose dasatinib treatment prevents the development of HCM in NSML mice.

### Transcriptome Analysis in the Hearts of Low-dose Dasatinib-treated NSML Mice

The above experiments showed low-dose dasatinib treatment prevents the progression of HCM in NSML mice (Figs. 3, 4, and 5 and Table 2). In order to test whether low-dose dasatinib also could reverse established HCM, we treated NSML mice with low-dose dasatinib at the age of 14 weeks which presents with prominent HCM. WT or NSML mice at 14 weeks old were treated with either vehicle or 0.1 mg/kg of dasatinib for 4 weeks (Supplementary Fig. 1a). We found that HW to BW ratio (HW/BW) was significantly reduced in dasatinib-treated NSML mice compared with vehicle-treated NSML mice (Supplementary Fig. 1b-d). These data indicate that a low-dose dasatinib reverses and/or slows the progression of HCM in NSML mice.

**Fig. 6:**
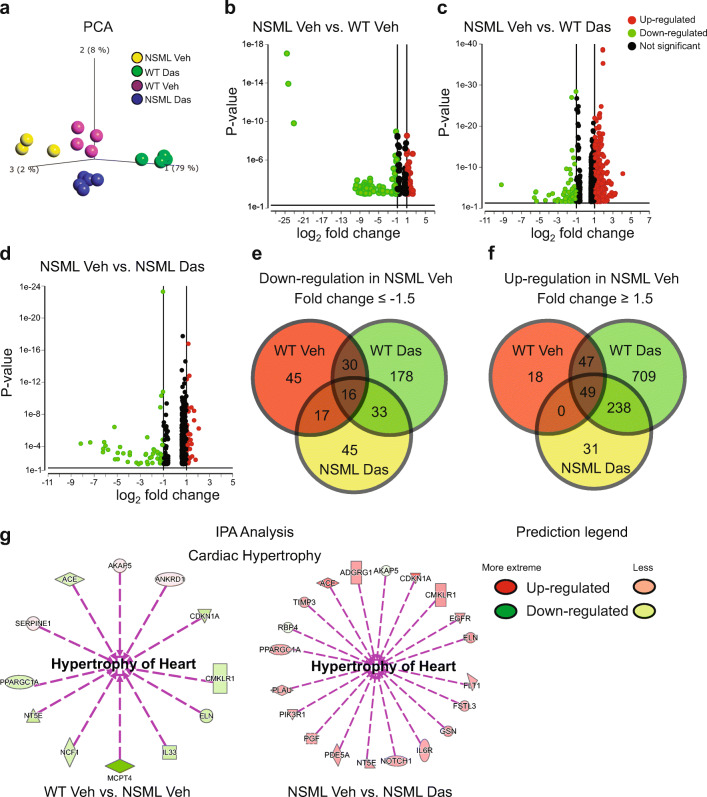
RNA-seq whole transcriptome analysis of low-dose dasatinib-treated NSML mice. **a** Principal component (PCA) analysis plot displaying all 4 groups along PC1, PC2, and PC3 which describe 79%, 8%, and 2% of the variability, respectively. PCA analysis (*p* = 0.0001) was applied to normalized counts and log-transformed count data. **b**, **c**, **d** Volcano plot of differentially expressed genes in (b) vehicle-treated NSML vs. vehicle-treated WT, (c) vehicle-treated NSML vs. dasatinib-treated WT, and (d) vehicle-treated NSML vs. dasatinib-treated NSML. The expression difference is considered significant for a fold change ≤ − 2.0 or ≥ 2.0, and for a *p* ≤ 0.05 and FDR step up ≤0.1. Downregulated genes are shown in green and upregulated genes are shown in red. **e**, **f **Venn diagram representation showing the number of (e) down- and (f) upregulated DEGs in vehicle-treated NSML compared with vehicle-treated WT, dasatinib-treated WT, and dasatinib-treated NSML. **g** Ingenuity pathway analysis (IPA) performed on statistically significant genes (*p* < 0.05) to identify low-dose dasatinib-responsive genes involved in cardiac hypertrophy.

Next, we sought to obtain further insight into the actions of low-dose dasatinib in reversing HCM in NSML mice by performing whole-transcriptome RNA-sequencing (RNA-Seq) analysis in vehicle or dasatinib-treated wild-type (WT) and NSML mice. Principal component analysis (PCA) showed that each treatment group clustered, suggesting that transcriptome profiles were significantly different in all groups (Fig. 6a). The hierarchical clustering of log ratio–transformed gene expression was represented in a heatmap and showed 4550 genes were differentially expressed between these four groups (Supplementary Fig. 2).

In order to identify low-dose dasatinib-specific target genes in the hearts of NSML mice we performed DESeq-2 to identify differentially expressed genes (DEGs). This analysis showed that 122 genes were downregulated and 129 genes were upregulated in the hearts of vehicle-treated NSML mice compared with vehicle-treated WT mice (Fig. 6b). When vehicle-treated NSML mice were compared with dasatinib-treated WT mice, 298 and 1086 genes were downregulated and upregulated, respectively (Fig. 6c). One hundred and twenty-five genes were found to be downregulated and 322 genes were upregulated in the vehicle-treated NSML mice compared with dasatinib-treated NSML mice (Fig. 6d). With these DEG profiles, we further analyzed commonly upregulated or downregulated genes in the heart of vehicle-treated NSML mice compared with the other three treatment conditions. (Fig. 6e and f). We observed that 16 genes were commonly downregulated (Fig. 6e, Supplementary Table 3) and 49 genes were commonly upregulated (Fig. 6f, Supplementary Table 4) in the hearts of vehicle-treated NSML mice compared with the other three groups. Ingenuity pathway analysis (IPA) identified cardiac hypertrophy-associated genes, such as peroxisome proliferator-activated receptor gamma coactivator 1 alpha (*Ppargc1a*), Elastin (*Eln*), angiotensin-I-converting enzyme (*Ace*), 5’-nucleotidase (*Nt5e*), cyclin-dependent kinase inhibitor 1A (*Cdkn1a*), chemerin chemokine-like receptor 1 (*Cmklr1*), and A-kinase anchor protein 5 (*Akap5*) gene was differentially expressed in the hearts of vehicle-treated NSML mice compared to vehicle-treated WT mice (Fig. 6g). Moreover, these genes were reversed in their expression after low-dose dasatinib treatment in the heart of NSML mice (Fig. 6g). These transcriptome analyses demonstrate that low-dose dasatinib exerts transcriptional changes in the hearts of NSML mice. Furthermore, a defined sub-set of target genes have been identified that are relevant to HCM and reverted in a dasatinib-dependent manner providing mechanistic insight into the actions of dasatinib in HCM reversal in NSML mice.

### Gene Ontology Enrichment Analysis of Differential Expressed Genes in Low-dose NSML-treated Mice

**Fig. 7:**
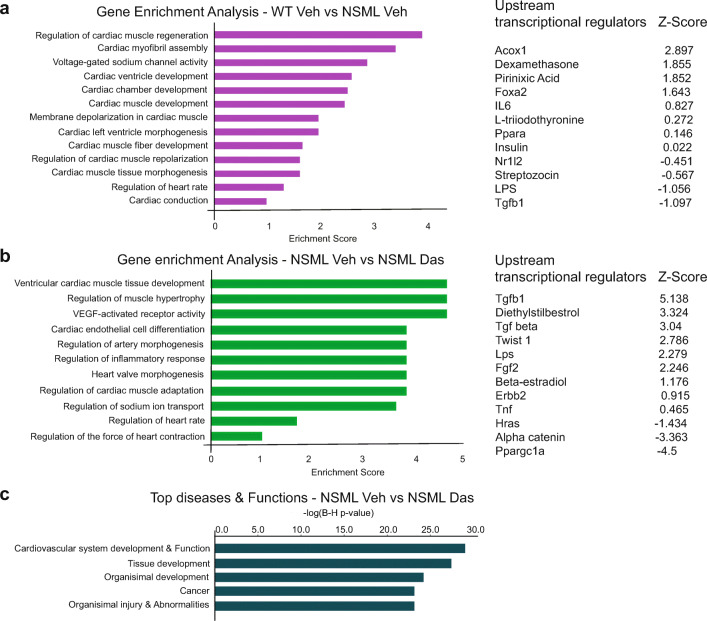
Gene ontology analysis of low-dose dasatinib-treated NSML mice. **a**, **b** Gene ontology (GO) enrichment analysis was performed from the gene list of (a) vehicle-treated WT vs. vehicle-treated NSML and (b) vehicle-treated NSML vs. dasatinib-treated NSML comparisons. Top biological processes were ranked by enrichment score. Upstream transcription regulators were listed. **c** Top 5 diseases and biological functions were analyzed from the differentially expressed gene list of vehicle-treated NSML vs. dasatinib-treated NSML comparisons.

To identify characteristic biological processes driving HCM in NSML mice and to determine if those pathways were dasatinib-sensitive, we performed gene ontology (GO) enrichment analysis (Fig. 7a, b). The top GO term–ranked biological processes in vehicle-treated WT compared to vehicle-treated NSML mice were cardiac muscle regeneration, cardiac myofibril assembly, voltage-gated sodium channel activity, cardiac ventricle development, and cardiac chamber development (Fig. 7a). The top-ranked biological processes in vehicle-treated NSML vs. dasatinib-treated NSML comparison were ventricular cardiac muscle tissue development, regulation of muscle hypertrophy, Vegf-activated receptor activity, cardiac endothelial cell differentiation, regulation of artery morphogenesis, and regulators of inflammatory responses (Fig. 7b). We also identified the top five diseases in vehicle-treated NSML vs. dasatinib-treated NSML comparison. Gratifyingly, this analysis revealed cardiovascular system development and function as one of the top ranked diseases corrected by dasatinib treatment (Fig. 7c). Furthermore, we investigated upstream regulators by IPA to identify the cascade of upstream transcriptional regulators that might explain the observed gene expression changes in our dataset, which could shed light on the biological activities occurring in the heart of low-dose dasatinib-treated NSML mice. The upstream regulators are ranked by activation *Z*-score. The upstream transcriptional regulators in WT vehicle vs. NSML vehicle comparison include, *Foxa*, *Il6*, *Ppara*, *Insulin*, *Nr1l2*, and *Tgfβ1* (Fig. 7a). In NSML vehicle vs. NSML dasatinib comparison, the upstream transcriptional regulators were found to include β-estradiol, *Twist1*, *Ppargc1a*, *Hras*, *Tnf*, *Fgf2*, and *Tgfb1* (Fig. 7b). Interestingly, some of these targets such as *Tgfb1*, *Hras*, *Fgf2*, and *Ppargc1a* have been implicated in cardiac hypertrophy suggesting that low-dose dasatinib indeed impacts genes that affect heart growth and remodeling [33–40].

## Discussion

NSML is a very rare genetic disorder with one of the major life-threatening presentations being the development of HCM in over 85% of these patients [18]. HCM is characterized by ventricular hypertrophy, impaired diastolic function, and an increased risk of sudden death [41]. NSML-associated *PTPN11* mutations have been shown to hyperactivate the AKT/mTOR pathway, and are believed to be one of the operative mechanisms promoting pathophysiological growth of the myocardium [27, 28, 42]. Therefore, development of inhibitors targeting the AKT/mTOR pathway has been proposed as a therapy for the treatment of HCM in NSML patients. As such, some limited success has been reported in the treatment of HCM in a NSML patient with the mTOR inhibitor, Everolimus [29]. Indeed, the repurposing of cancer drugs as an early entry therapeutic path for the treatment of rare diseases is an attractive proposition from both a safety and rapid development perspective for an unmet medical need [43]. We previously suggested that a low dose of dasatinib could provide a therapeutic avenue for the treatment of cardiomyopathies in both NS and NSML [21]. Although low-dose dasatinib was shown to be effective in improving cardiac function in NS, this study did not directly address the effects of low-dose dasatinib in the development of NSML-associated HCM [21]. We now show that low-dose dasatinib treatment is effective in ameliorating HCM in NSML mice.

Dasatinib is a small molecule inhibitor developed for the treatment of CML and acute lymphoblastic leukemia (ALL) in patients who are positive for the Philadelphia chromosome [44, 45]. In a mouse model, initial studies demonstrated that 10 mg/kg administered twice daily of dasatnib inhibits the growth of murine hematopoietic Ba/F3 cancer cells in SCID mice, and 2.5 mg/kg daily administration was sufficient to inhibit imatinib-resistant K562/R tumor growth [30, 46]. Whereas, the clinical doses of dasatinib can cause several adverse effects, such as hemorrhage and QT prolongation [47], we showed 0.1 mg/kg daily administration of dasatinib, which is up to 200-fold lower in dose compared to that used for the treatment of CML, displayed no apparent toxicity in mice [21]. Importantly, postnatal administration of dasatinib (0.1 mg/kg) in NSML mice prevented the development of HCM and myocardial fibrosis [21]. At such low doses of dasatinib, it was important to define the pharmacokinetics and the pharmacodynamics of this drug in mice. Our study revealed that dasatinib clearance after intraperitoneal administration was comparable to the clearance estimate reported in mice receiving 5.0 mg/kg as an intravenous injection [30]. Our exposure-response analysis indicated that the area under the plasma dasatinib concentration-time curve required to inhibit 50% (IAUC_50_) of c-Src phosphorylation at its activating Y416 site was 2.1 ± 3.6 ng·h/mL representing a dose of dasatinib of 0.007 mg/kg. The IAUC_50_ for PZR tyrosyl phosphorylation at residues 242 and 264 were 59 ng·h/mL and 19 ng·h/mL, which can be achieved at a dose level of 0.24 mg/kg and 0.07 mg/kg, respectively. Thus, PZR tyrosyl phosphorylation and c-Src, the likely tyrosine kinase in which it is phosphorylated by, are inhibited at this low dose of dasatinib. These data further support the notion that dasatinib can be used at highly desirable low doses to engage its target(s) and concomitantly markedly reduce toxicities associated with chemotherapeutic levels that have been reported to cause cardiac toxicity in humans. It has previously been reported that a dose of dasatinib that is ~50-fold lower than that used for chemotherapeutic purposes also inhibits non-receptor tyrosine kinases such as Pyk2 and focal adhesion kinase in mice [48]. Furthermore, at these lower doses, dasatinib improved cardiac functionality in a mouse model of pressure overload [48]. Together with our results, these data demonstrate that low doses of dasatinib may have clinical utility. The PK and PD analyses presented here serve as important pre-clinical data to support derivation of the corresponding dose of dasatinib to treat NSML patients for HCM.

AKT/mTOR signaling is a crucial positive regulator of cardiac tissue mass [25, 49]. In the heart, AKT is activated under conditions of pressure overload and genetic disorders, promoting pathophysiological cardiac hypertrophy [24, 25]. AKT phosphorylation increases the activity of mTOR substrates enhancing protein synthesis [50, 51]. AKT also has protective effects on apoptosis and muscle atrophy by negatively regulating downstream targets [52]. Previous work has shown that AKT/mTOR signaling is increased in the heart of NSML mice, suggesting this pathway is involved in the pathogenesis of HCM in NSML [27, 42, 53]. Although insulin increases AKT phosphorylation in cardiomyocytes isolated from NSML mice, physiological insulin serum levels in NSML mice are significantly lower compared to WT mice suggesting that insulin action is unlikely to be responsible for the increased levels of AKT activity in NSML mice [54]. As such, the precise mechanisms driving AKT hyperactivation in NSML still remains to be fully defined. Marin et al. suggested excessive focal adhesion kinase (FAK) activity may be involved in PI3K/AKT activation in NSML [27]. It has been reported that genetic or chemical inhibition of FAK decreases AKT activation in various animal models of cardiomyopathy [55, 56]. Heart lysates isolated from NSML mice have elevated FAK tyrosyl phosphorylation at Y397 [27]. Tyrosine 397 of FAK is phosphorylated by c-Src kinase which can engage in cross-talk with the AKT pathway [57]. However, how NSML-associated SHP2 mutations induce FAK tyrosyl phosphorylation is unclear. Interestingly, tumor necrosis factor-α (TNF-α) promotes cardiomyocyte growth and can activate AKT [58] and we have shown that this cytokine is increased in NSML mice [22]. It remains to be determined whether TNF-α drives NSML-associated AKT activation and if so whether it is inhibited by dasatinib.

We have found that PZR is increased in its level of tyrosyl phosphorylation in heart lysates of NSML mice and this correlates with increased AKT activation and expression of HCM-associated genes, such as *Myh7*, *Nppa*, *Nppb*, and *Col1a* [22]. When a phosphorylation-defective PZR mutation (PZR^Y242F^) was introduced into NSML mice, we found that HCM phenotypes of NSML mice were reversed [22]. These results indicate that PZR tyrosyl phosphorylation contribute to the activation of HCM-related pathway(s) in NSML. In light of these results and others, we proposed that NSML-associated SHP2 mutations increases the binding affinity to both PZR and Src family kinases (SFKs), thereby promoting the accumulation of a complex comprising of PZR/SHP2/SFK that promotes HCM in NSML mice [20–22]. Treatment of low-dose dasatinib in NSML mice reverses HCM-related pathways that correlate with the inhibition of PZR tyrosyl phosphorylation and PZR/SHP2 interaction which could serve to be pathological drivers of HCM. It is not unreasonable to suggest that low-dose dasatinib, at least in-part, prevents the development of HCM in NSML by this mechanism. This study does not preclude the other effects of dasatinib that are also likely to prevent the development of HCM in NSML. Nevertheless, the ability to inhibit AKT activity and HCM-associated gene expression in NSML mice at such low doses of dasatinib strongly suggests that its action at this dose engages targets that are known to directly regulate HCM progression.

HCM is typically characterized by a hypertrophied and non-dilated left ventricle that is accompanied by normal to hyperdynamic functionality of left ventricular function during systole. However, around 8% of HCM patients have been reported to exhibit left ventricular systolic dysfunction [59]. HCM patients with pathogenic sarcomeric variants have a higher risk of developing HCM with left ventricular systolic dysfunction. Interestingly, NSML patient’s with early onset obstructive HCM have shown a reduced ejection fraction also suggesting left ventricular dysfunction [29, 60]. Here, we observed that the increased left ventricular wall thickness and reduced functionality in NSML mice were normalized after low-dose dasatinib treatment demonstrating that 0.1 mg/kg dose of dasatinib can prevent the onset of this pathological event. The effects of dasatinib on interventricular septum wall thickness and left ventricular functionality were modest, but given that the period of low-dose dasatinib treatment occurred only over a 4-week time period it is conceivable that more prolonged treatment could produce more profound effects.

Several studies have uncovered abnormal HCM signaling pathways in NSML, but little is known about the alterations of the cardiac transcriptome induced by NSML-SHP2 mutations. Hence, to gain insights into the transcriptional profiles which are induced by the NSML-SHP2 mutation and those that are reversed by low-dose dasatinib treatment in NSML mice, we performed quantitative transcriptome profiling by RNA-sequencing in vehicle- and dasatinib-treated NSML mice. In particular, our RNA sequencing data suggest that dasatinib reverses the expression of the cardiomyocyte regeneration gene, *Cdkn1a*, in the heart of NSML mice. *Cdkn1a* encodes for the protein cyclin–dependent kinase inhibitor 1A, also known as p21. p21 is associated with cell cycle regulation. *Cdkn1a* expression is increased in cardiomyocytes during the fetal-to-neonatal transition resulting in withdrawal of cardiomyocytes from the cell cycle [61, 62]. It has been reported that *Cdkn1a* is upregulated in hypertrophic cardiomyocytes or in cardiomyopathic heart tissue [63, 64]. Notably, NSML mice express *Cdkn1a* in both valves and myocardium through AKT-mediated down-regulation of FOXP activity [42].

Our gene ontology pathway analysis defined cardiac muscle development and hypertrophic genes as significantly involved when vehicle-treated NSML mice were compared to vehicle-treated WT mice or dasatinib-treated NSML mice. We also showed that the transforming growth factor-β (TGFβ) is one of the top-ranked upstream transcriptional regulators in NSML mice. TGFβ signaling is known to play an important role in the pathogenesis of cardiac remodeling and interstial myocardial fibrosis [33, 65]. TGFβ expression is increased in animal models of myocardial infarction or HCM and in human patients with HCM [66, 67]. Further work will be necessary to validate and fully assign a causal relationship between the expression of these genes and low-dose dasatinib effects. Nevertheless, genome-wide RNA-sequencing analysis identifies several pathways relating to cardiac hypertrophy and remodeling that are targets for reversal by low-dose dasatinib treatment. The identity of the exact target or, more likely targets, that are directly or indirectly affected by dasatinib at this low dose to cause the reversal of HCM development in NSML and NS remains to be determined.

In summary, the results presented herein demonstrate that low-dose dasatinib is effective at preventing the development of HCM in a mouse model of NSML. NS and NSML represent mutations in SHP2 that render opposing effects on its catalytic activity and as such unifying therapies that target SHP2 catalysis are unlikely to be effective in the treatment of these diseases [10–12]. The implications of this work that now in addition to NS, NSML also is responsive to low-dose dasatinib suggest that both forms of these RASopathies are equally amenable to be considered for treatment against the development of HCM using this strategy. As such, low-dose dasatinib treatment should be considered as a unifying therapeutic strategy for all *PTPN11*-associated SHP2 mutations that cause reduced cardiac function and HCM in children.

## Supplementary Information

ESM 1 (DOCX 441 kb)

## Data Availability

Most of the data described in the article are contained within the article or in the supporting information. Further information and requests for resources and reagents should be directed to and will be fulfilled by Anton M. Bennett (anton.bennett@yale.edu) and Jae-Sung Yi (jae-sung.yi@yale.edu).

## Abbreviations

CHD: Congenital heart disease
HCM: Hypertrophic cardiomyopathy
NS: Noonan syndrome
NSML: Noonan syndrome with multiple lentigines
PK/PD: Pharmacokinetics/Pharmacodynamics
PTPs: Protein tyrosine phosphatases
PZR: Protein zero-related
SFKs: Src family kinases
SHP2: Src homology 2 domain-containing protein tyrosine phosphatase 2
