# Supplementary material for: Low-dose Dasatinib Ameliorates Hypertrophic Cardiomyopathy in Noonan Syndrome with Multiple Lentigines
Source: Cardiovasc Drugs Ther. 2021 Mar 10;36(4):589–604. doi: 10.1007/s10557-021-07169-z (PMC9270274; doi:10.1007/s10557-021-07169-z)
Supplement: Supplementary file 1 — (DOCX 441 kb) [file 10557_2021_7169_MOESM1_ESM.docx]

**Article Title**: Low dose dasatinib ameliorates hypertrophic cardiomyopathy in Noonan syndrome with multiple lentigines

**Journal Name**: Cardiovascular Drugs and Therapy

**Authors**: Jae-Sung Yi^1,*^, Sravan Perla^1^, Yan Huang^2^, Kana Mizuno^4^, Frank J. Giordano^2^, Alexander A. Vinks^4,5^ and Anton M. Bennett^1,3^

^1^Department of Pharmacology, ^2^Department of Internal Medicine and ^3^Program in Integrative Cell Signaling and Neurobiology of Metabolism, Yale University School of Medicine, New Haven, CT, 06520, USA, ^4^Division of Clinical Pharmacology, Cincinnati Children's Hospital Medical Center, Cincinnati, OH, USA, ^5^Department of Pediatrics, University of Cincinnati, College of Medicine, Cincinnati, OH, USA.

^*^Corresponding author : Jae-Sung Yi

Department of Phamacology

Yale University School of Medicine

Sterling Hall of Medicine, B225

333 Cedar Street, New Haven,

Connecticut, 06520-8066

E-mail: [jae-sung.yi@yale.edu](mailto:jae-sung.yi@yale.edu)

**Supplementary Fig. 1. Low dose dasatinib reverses HCM in NSML mice.** **(a)** Schematic of low dose dasatinib administration. Vehicle or 0.1 mg/kg of dasatinib was intraperitoneally (i.p., daily) injected into 14-weeks-old WT or NSML mice for 4 weeks. At the age of 18-weeks, heart tissues were isolated and were subjected to whole transcriptomic RNA-seq analysis. Body weight (BW) **(b)**, heart weight (HW) **(c)** and the ratio of HW to BW (HW/BW) **(d)** were measured from Vehicle or 0.1 mg/kg of dasatinib treated WT and NSML mice. (*n* = 7 for vehicle-treated WT and NSML mice, *n* = 5 for dasatinib-treated WT and NSML mice). Data represent mean ± SEM. 2-way ANOVA with 2-stage linear step-up procedure of Benjamini, Krieger, and Yekutieli correction for multiple comparisons.

**Supplementary Fig. 2**. **Hierarchical clustering analysis of the relative gene expression of vehicle- or low dose dasatinib treated WT and NSML mice.** Hierarchical clustered heatmap of log2-transformed gene expression in the hearts of vehicle- or low dose dasatinib treated WT and NSML mice (*p* = 0.01). Each column represents an individual mouse and each row represents a gene. Differential gene expression was shown in the heatmap. The blue color indicated lower gene expression level and the red showed higher gene expression level.

**Supplementary Table 1. The list of primer sequences for quantitative real-time PCR analysis**

| Primer Name | Sequences |
| --- | --- |
| 18S rRNA | 5’-ACCGCAGCTAGGAATAATGGA-3’  5’-GCCTCAGTTCCGAAAACCA-3’ |
| *Myh6* | 5’-GTCCCGGACACTGGACCAGGCC-3’  5’-CTCCTTTTCTTCCAGTTGCCTAGCCAA-3’ |
| *Myh7* | 5’-GAGCAAGGCCGAGGAGACGCAGCGT-3’  5’-GAGCCTCCTTCTCGTCCAGCTGCCGG-3’ |
| *Nppa* | 5’- CCTGGAGGAGAAGATGCCGGTAGAA-3’  5’-CCCCAGTCCAGGGAGGCACCTCGG-3’ |
| *Nppb* | 5’-CACTTCAAAGGTGGTCCCAGAGCTGC-3’  5’-GACCGGATCGGATCCGTCAGTCG-3’ |
| *Col1a* | 5’-AGGTCTTCCTGGAGCTGATG-3’  5’-ACCCACAGGGCCTTCTTTAC-3’ |
| *Col3a* | 5’-ACAGCAAATTCACTTACACAGTTC-3’  5’-CTCATTGCCTTGCGTGTTT-3’ |

**Supplementary Table 2. Echocardiography parameters of vehicle- and 0.1 mg/kg dasatinib (i.p., daily)-treated WT and NSML mice.** Data represent mean ± SEM (*n* = 6). ^*^, *p* < 0.05; ^**^, *p* < 0.01; ^***^, *p* < 0.001 denotes significance compared to the vehicle treated WT mice. ^†^, *p* < 0.05; ^††^, *p* < 0.01; ^†††^, *p* < 0.001 denotes significance compared to the vehicle-treated NSML mice. ^##^, *p* < 0.01 denotes significance compared to the dasatinib-treated WT mice. 2-way ANOVA with 2-stage linear step-up procedure of Benjamini, Krieger, and Yekutieli correction for multiple comparisons. IVS, Interventricular septum wall thickness; LVID, left ventricular internal dimension; LVPW, left ventricular posterior wall thick ness; LV vol, left ventricle volume; LV mass, left ventricle mass; %EF, percentage of ejection fraction; %FS, percent of fractional shortening; d, diastolic phase; s, systolic phase.

|  | WT Vehicle | NSML Vehicle | WT Dasatinib | NSML Dasatinib |
| --- | --- | --- | --- | --- |
| IVS,d (mm) | 0.65±0.02 | 0.86±0.02^***^ | 0.57±0.04^†††^ | 0.72±0.03^††,##^ |
| IVS,s (mm) | 1.02±0.06 | 1.16±0.03 | 0.95±0.05^††^ | 0.99±0.03^†^ |
| LVID,d (mm) | 3.96±0.11 | 3.90±0.12 | 3.93±0.11 | 4.05±0.10 |
| LVID,s (mm) | 2.66±0.05 | 3.04±0.12^*^ | 2.98±0.16 | 2.98±0.07 |
| LVPW,d (mm) | 0.72±0.02 | 0.91±0.04^**^ | 0.75±0.05^††^ | 0.78±0.03^†^ |
| LVPW,s (mm) | 1.10±0.06 | 1.09±0.04 | 1.14±0.06 | 1.08±0.04 |
| LV vol,d (mm^3^) | 69.85±4.11 | 66.50±4.70 | 78.93±5.84 | 69.51±2.73 |
| LV vol,s (mm^3^) | 25.86±1.09 | 36.72±3.73^*^ | 35.20±4.51^*^ | 34.59±1.83 |
| LV mass (mm^3^) | 97.22±4.27 | 129.39±6.84^***^ | 106.07±6.41^††^ | 110.06±3.04^†^ |
| %EF | 62.50±2.27 | 45.31±1.74^***^ | 56.13±3.00^†^ | 50.27±1.62^**^ |
| %FS | 33.42±1.69 | 22.18±0.95^***^ | 31.87±2.06^†††^ | 25.20±0.99^**^ |

**Supplementary Table 3.** **The list of commonly down-regulated genes in the heart of vehicle treated NSML mice represented in Fig. 5E.** FDR, false discovery rate; FC, fold change.

|  | NSML Veh vs.  WT Veh | | NSML Veh vs.  WT Das | | NSML Veh vs.  NSML Das | |
| --- | --- | --- | --- | --- | --- | --- |
| Gene Symbol | FDR | FC | FDR | FC | FDR | FC |
| *Akap5* | 3.57E-02 | -2.225 | 2.62E-08 | -3.259 | 2.35E-03 | -1.942 |
| *Cpxm2* | 1.45E-02 | -1.544 | 1.77E-06 | -1.891 | 8.63E-05 | -1.712 |
| *Fgf9* | 1.88E-03 | -1.866 | 4.93E-06 | -1.982 | 2.90E-03 | -1.676 |
| *Gm3222* | 6.86E-02 | -2.322 | 4.49E-02 | -2.253 | 5.81E-02 | -2.164 |
| *Gm11633* | 8.05E-02 | -2.061 | 5.77E-02 | -2.022 | 6.02E-02 | -1.926 |
| *Gm19410* | 6.99E-02 | -1.856 | 2.31E-02 | -1.947 | 6.59E-02 | -1.680 |
| *Gsta1* | 2.56E-02 | -1.747 | 4.08E-03 | -1.906 | 1.08E-02 | -1.785 |
| *Nr1d1* | 7.65E-02 | -2.181 | 4.90E-06 | -3.012 | 4.68E-08 | -2.271 |
| *Pck1* | 1.32E-02 | -113.116 | 5.65E-02 | -20.853 | 3.40E-03 | -27.091 |
| *Rn7s1* | 7.62E-02 | -2.152 | 1.81E-09 | -2.273 | 2.47E-04 | -1.696 |
| *Rn7s2* | 3.25E-06 | -2.411 | 3.24E-24 | -2.850 | 1.74E-08 | -2.048 |
| *Serpinc1* | 3.98E-02 | -86.616 | 1.81E-02 | -48.401 | 4.49E-05 | -37.942 |
| *Srrm4* | 5.90E-04 | -2.181 | 1.59E-12 | -2.772 | 1.89E-02 | -1.700 |
| *Tnfrsf19* | 6.08E-02 | -1.770 | 1.39E-03 | -2.021 | 3.39E-05 | -1.963 |
| *Ucp3* | 3.49E-02 | -2.822 | 3.85E-02 | -1.904 | 1.71E-02 | -2.298 |
| *Zim1* | 5.59E-02 | -1.939 | 3.79E-04 | -2.727 | 2.78E-02 | -1.836 |

**Supplementary Table 4. The list of commonly up-regulated genes in the heart of vehicle treated NSML mice represented in Fig. 5F.** FDR, false discovery rate; FC, fold change.

|  | NSML Veh vs.  WT Veh | | NSML Veh vs.  WT Das | | NSML Veh vs.  NSML Das | |
| --- | --- | --- | --- | --- | --- | --- |
| Gene Symbol | FDR | FC | FDR | FC | FDR | FC |
| *Abca8a* | 8.24E-02 | 1.552 | 1.05E-20 | 2.971 | 2.21E-04 | 1.667 |
| *Abi3bp* | 7.48E-02 | 1.896 | 1.28E-08 | 3.208 | 1.92E-03 | 1.933 |
| *Ace* | 1.28E-02 | 1.695 | 4.06E-09 | 3.746 | 7.22E-14 | 2.239 |
| *Adgrg6* | 3.28E-02 | 2.555 | 1.13E-07 | 5.272 | 1.80E-03 | 2.725 |
| *Aebp1* | 5.01E-02 | 1.757 | 1.25E-11 | 3.554 | 4.10E-05 | 1.913 |
| *Angptl7* | 9.18E-02 | 3.491 | 1.92E-03 | 7.316 | 6.23E-05 | 4.181 |
| *Axl* | 3.05E-02 | 1.555 | 1.86E-17 | 2.773 | 5.76E-07 | 1.778 |
| *Baiap2* | 1.81E-02 | 2.152 | 3.12E-04 | 2.331 | 1.91E-02 | 1.596 |
| *Bcl2l1* | 1.67E-02 | 1.771 | 8.71E-18 | 2.807 | 1.10E-07 | 1.777 |
| *Cdh19* | 8.24E-02 | 1.972 | 4.15E-09 | 3.269 | 6.29E-03 | 1.971 |
| *Cdkn1a* | 5.44E-03 | 2.095 | 3.52E-08 | 2.207 | 3.02E-10 | 2.377 |
| *Cmklr1* | 4.53E-02 | 1.643 | 1.69E-15 | 2.965 | 9.43E-05 | 1.732 |
| *Cntfr* | 5.73E-02 | 2.374 | 9.61E-05 | 3.365 | 3.36E-02 | 1.712 |
| *Col28a1* | 5.74E-03 | 3.309 | 1.89E-05 | 5.221 | 9.54E-06 | 3.161 |
| *Cxcl14* | 5.74E-03 | 1.951 | 8.30E-10 | 2.623 | 3.47E-04 | 1.645 |
| *Efemp1* | 1.45E-02 | 1.864 | 1.70E-11 | 3.465 | 5.76E-07 | 2.096 |
| *Eln* | 5.99E-03 | 1.784 | 1.67E-05 | 2.009 | 1.73E-04 | 1.834 |
| *Fkbp5* | 9.89E-02 | 2.451 | 1.12E-03 | 3.957 | 2.33E-04 | 2.510 |
| *Gdf10* | 3.96E-02 | 2.308 | 1.09E-04 | 3.519 | 6.09E-04 | 2.208 |
| *Glt28d2* | 7.61E-02 | 1.690 | 2.53E-03 | 1.648 | 2.06E-02 | 1.672 |
| *Hhipl1* | 4.75E-02 | 2.088 | 1.27E-09 | 4.871 | 4.10E-05 | 1.992 |
| *Htra3* | 6.86E-02 | 1.540 | 9.16E-20 | 2.905 | 3.67E-06 | 1.764 |
| *Ifi205* | 2.49E-03 | 2.274 | 2.78E-05 | 3.838 | 4.26E-06 | 1.794 |
| *Iqgap2* | 2.33E-02 | 1.645 | 2.56E-04 | 1.956 | 2.21E-02 | 1.552 |
| *Kcnk6* | 3.47E-02 | 1.521 | 2.64E-06 | 2.065 | 1.16E-04 | 1.750 |
| *D630003M21Rik* | 6.88E-02 | 1.665 | 3.05E-09 | 2.818 | 2.84E-02 | 1.574 |
| *L1cam* | 2.94E-02 | 1.936 | 1.67E-05 | 2.618 | 4.19E-03 | 1.657 |
| *Lvrn* | 4.66E-02 | 3.503 | 2.11E-03 | 3.688 | 3.17E-02 | 2.263 |
| *Lyve1* | 5.81E-06 | 2.067 | 2.60E-35 | 3.714 | 6.35E-10 | 1.808 |
| *Map3k6* | 9.30E-02 | 2.404 | 1.88E-05 | 5.565 | 6.49E-04 | 1.972 |
| *Matn2* | 4.95E-02 | 1.712 | 4.17E-16 | 3.735 | 2.95E-05 | 2.063 |
| *Mmp19* | 3.43E-02 | 2.091 | 4.06E-04 | 2.512 | 3.28E-02 | 1.645 |
| *Ms4a6c* | 1.56E-03 | 2.299 | 1.39E-09 | 2.795 | 6.93E-02 | 1.616 |
| *Nfil3* | 1.32E-02 | 2.058 | 4.03E-05 | 1.859 | 2.50E-03 | 2.009 |
| *Nmb* | 2.51E-03 | 2.564 | 5.91E-10 | 5.339 | 5.76E-07 | 2.694 |
| *Nt5e* | 1.13E-04 | 2.094 | 3.57E-06 | 1.819 | 5.86E-03 | 1.665 |
| *Pdgfra* | 5.75E-02 | 1.561 | 3.00E-16 | 2.760 | 7.09E-07 | 1.856 |
| *Podn* | 3.08E-02 | 1.717 | 2.56E-13 | 2.491 | 3.30E-03 | 1.781 |
| *Ppargc1a* | 3.99E-02 | 1.605 | 2.43E-08 | 1.572 | 1.63E-03 | 1.628 |
| *Prom1* | 8.87E-02 | 2.169 | 1.62E-02 | 2.490 | 7.41E-02 | 1.926 |
| *Rab27a* | 7.61E-02 | 1.901 | 4.05E-08 | 3.150 | 5.24E-05 | 1.999 |
| *Rin3* | 2.00E-02 | 1.648 | 1.57E-14 | 2.632 | 8.52E-12 | 1.875 |
| *Rtn4rl2* | 9.91E-02 | 2.239 | 9.71E-06 | 6.104 | 7.64E-04 | 2.569 |
| *Sh3pxd2b* | 1.71E-02 | 1.644 | 2.31E-06 | 2.144 | 9.96E-07 | 1.966 |
| *Slc10a6* | 7.49E-02 | 2.131 | 2.51E-07 | 4.937 | 7.18E-03 | 1.723 |
| *Slc43a3* | 3.98E-02 | 1.862 | 1.44E-05 | 3.108 | 1.34E-06 | 1.691 |
| *Spon1* | 6.86E-02 | 1.678 | 3.17E-04 | 3.078 | 3.91E-05 | 1.958 |
| *Tlr7* | 3.07E-02 | 1.842 | 6.21E-07 | 2.416 | 5.33E-02 | 1.606 |
| *Tmem255a* | 2.62E-02 | 2.626 | 2.00E-02 | 2.667 | 4.70E-02 | 1.895 |
